# Extended UVB Exposures Alter Tumorigenesis and Treatment Efficacy in a Murine Model of Cutaneous Squamous Cell Carcinoma

**DOI:** 10.1155/2013/246848

**Published:** 2013-10-27

**Authors:** Erin M. Burns, Kathleen L. Tober, Judith A. Riggenbach, Donna F. Kusewitt, Gregory S. Young, Tatiana M. Oberyszyn

**Affiliations:** ^1^Department of Pathology, The Ohio State University, 1645 Neil Avenue, 129 Hamilton Hall, Columbus, OH 43210, USA; ^2^Department of Molecular Carcinogenesis, Science Park, UT MD Anderson Cancer Center, 1808 Park Road 1C, Smithville, TX 78957, USA; ^3^Center for Biostatistics, The Ohio State University, 2012 Kenny Road, Columbus, OH 43221, USA

## Abstract

Epidemiological studies support a link between cumulative sun exposure and cutaneous squamous cell carcinoma (SCC) development. However, the presumed effects of extended ultraviolet light B (UVB) exposure on tumorigenesis in the sexes have not been formally investigated. We examined differences in ultimate tumorigenesis at 25 weeks in mice exposed to UVB for either 10 or 25 weeks. Additionally, we investigated the effect of continued UVB exposure on the efficacy of topical treatment with anti-inflammatory (diclofenac) or antioxidant (C E Ferulic or vitamin E) compounds on modulating tumorigenesis. Vehicle-treated mice in the 25-week UVB exposure model exhibited an increased tumor burden and a higher percentage of malignant tumors compared to mice in the 10-week exposure model, which correlated with increases in total and mutant p53-positive epidermal cells. Only topical diclofenac decreased tumor number and burden in both sexes regardless of UVB exposure length. These data support the commonly assumed but not previously demonstrated fact that increased cumulative UVB exposure increases the risk of UVB-induced SCC development and can also affect therapeutic efficacies. Our study suggests that cessation of UVB exposure by at-risk patients may decrease tumor development and that topical NSAIDs such as diclofenac may be chemopreventive.

## 1. Introduction

 Epidemiological studies where patients self-report the amount of sun they have been exposed to over their lifetime have been the main source of the described link between cumulative, lifetime sunlight exposure and the development of cutaneous squamous cell carcinoma. While informative, with patients historically developing these lesions in their seventies, these self-reports may not accurately reflect the actual lifetime exposure history. Interestingly, we could not find any controlled studies reporting the effects of the length of lifetime UVB exposure on the extent of tumor development.

 Skin carcinogenesis experiments utilizing animal models, especially hairless mice, have contributed greatly to understanding how skin tumorigenesis depends on the wavelength of UV radiation, dose, and time [1]. The Skh-1 hairless mouse model has proven to be an appropriate and accepted model for experimental skin carcinogenesis [2]. Because the Skh-1 mice are hairless, tumors can be easily observed and their progression was tracked over time with relatively no discomfort for the mice. Importantly, after chronic UV exposure, shaved, haired mice can develop fibrosarcomas originating from the dermis [3], whereas the induction of tumors in hairless mice via chronic exposure of non-burn-inducing low UVB levels leads almost exclusively to epidermal squamous cell carcinoma and precursor development [4], which correlates to what is observed in UV-induced skin cancer in humans. 

 Skin tumors induced by chronic exposure to UV radiation progress from focal epithelial hyperplasia to papillomas and finally squamous cell and spindle cell carcinomas [4]. Previous studies in our laboratory using female mice have demonstrated that papilloma growth begins following 10–12 weeks of three times weekly UVB exposures. Approximately 90% of both male and female mice develop at least one tumor with a diameter greater than 1 mm after 16 weeks of three times weekly UVB exposures, with males having approximately 50% more tumors than females [5]. Squamous cell carcinoma development has been observed following 25–30 weeks of three times weekly UVB exposures [6], with males having more malignant tumors compared to females [5]. However, the effect of increased cumulative UVB exposure on tumorigenesis, while it presumed to increase in both sexes, has not been formally investigated.

 Previous murine studies have demonstrated that decreasing the daily UVB exposure dose results in the delay of tumor onset, indicating that patients at risk for developing cutaneous SCC may benefit from decreasing further UV exposure in order to inhibit or delay the development of tumors [7, 8]. Further, patients self-reporting high lifetime UV exposure had increased tumor multiplicity and severity compared to those self-reporting low lifetime UV exposure [9]. While these studies suggest that cumulative lifetime UV exposure may be correlated with the extent of cutaneous tumor development, no study to date has clearly demonstrated this relationship. Thus, we were interested in using the Skh-1 hairless mouse to model men and women who were exposed to UVB during childhood and early adolescence but made efforts to stay out of the sun in adulthood (10-week UVB exposure model) compared to individuals who continue sun worshiping habits throughout adult life (25-week UVB exposure model). Our goal was to determine if limiting the length of chronic UVB exposure (10 weeks versus 25 weeks) would affect the number of tumors that ultimately developed at 25 weeks.

Previously, we demonstrated that the topical anti-inflammatory drug, diclofenac, applied preventatively to chronically UVB-damaged skin of male and female mice, prior to the appearance of lesions, with no further UVB exposure, significantly decreased tumor number and burden compared to vehicle-treated, UVB-exposed mice [10]. We also previously demonstrated in female mice that while the combination antioxidant, C E Ferulic, exerted potential benefits in terms of decreased tumor number and burden, topical vitamin E treatment increased overall DNA damage, cutaneous proliferation and angiogenesis, and tumor growth rate, number, and burden [11]. In the current study, we compared the efficacy of anti-inflammatory (diclofenac) and antioxidant (C E Ferulic (CE) or vitamin E) treatments in male and female mice between the 10-week and 25-week UVB exposure models.

 Our results demonstrate that both male and female mice in the 25-week UVB exposure model developed more tumors, larger tumors, and a higher percentage of malignant tumors compared to mice in the 10-week UVB exposure model. Further, in the 25-week UVB exposure model only topical diclofenac treatments effectively decreased both the tumor number and total tumor area in male and female mice. These data demonstrate the commonly assumed fact that longer periods of UVB exposure increase the risk of UVB-induced SCC development in both sexes and suggest that eliminating sun exposure later in life, even after significant prior exposure, may ultimately decrease tumor development in patients. Furthermore, while treatment with topical diclofenac during continued UVB exposure was an effective chemopreventive agent, continued UVB exposure can negatively affect other therapeutic intervention strategies.

## 2. Materials and Methods

### 2.1. Animal Treatments and Experimental Design

Outbred, male and female Skh-1 mice (6–8 weeks old, Charles River Laboratories, Wilmington, MA) were housed in the vivarium at The Ohio State University according to the requirements established by the American Association for Accreditation of Laboratory Animal Care. All procedures were approved by the Institutional Animal Care and Use Committee before the initiation of any studies. Mice were dorsally exposed to 2240 J/m^2^ UVB, previously determined to be 1 MED, 3× weekly on nonconsecutive days for 10 (10-week UVB exposure model) or 25 (25-week UVB exposure model) weeks. UVB dose was calculated using a UVX radiometer and UVB sensor (UVP, Upland, CA) and delivered using Philips TL 40W/12 RS SLV UVB broadband bulbs emitting 290–315 nm UVB light (American Ultraviolet Company, Lebanon, IN). Following 10 weeks of UVB exposure, mice in the 10-week UVB exposure model were treated topically with vehicle (Surgilube inert surgical lubricant; Savage Laboratories, Melville, NY, *n* = 20 of each sex), 500 *μ*g diclofenac (Solaraze, *n* = 10 of each sex) in vehicle, 5 mg vitamin E (d-alpha tocopherol; Sigma-Aldrich, St. Louis, MO, *n* = 10 of each sex) in vehicle, or 0.1 mL C E Ferulic (SkinCeuticals, *n* = 10 of each sex) for 15 weeks with no additional UVB exposure. After 10 weeks of UVB exposure, mice in the 25-week UVB exposure model were treated topically with the aforementioned agents immediately following each UVB exposure for the remaining 15 weeks of the study. Tumors larger than 2 mm in diameter were counted and measured in two directions with calipers each week. Average tumor number per mouse, per treatment group was calculated for tumors larger than 2 mm in diameter. Tumor burden was calculated based on the average total tumor area per mouse, per treatment group. Mice in both the 10- and 25-week exposure models were sacrificed 25 weeks following the initial UV exposure. After sacrifice, 0.5 cm^2^ section of dorsal skin and all tumors were fixed in 10% neutral buffered formalin for 2 (skin) or 4 hours (tumors) while remaining dorsal skin was snap frozen in liquid nitrogen.

### 2.2. Tumor Grading

Hematoxylin and eosin-stained tissue sections of tumors isolated from mice were graded in a blinded manner by a board-certified veterinary pathologist (DFK) as previously described [5]. Briefly, papillomas were exophytic tumors (tumors that grow outward from the originating epithelium) that showed no invasion of the stroma. Microinvasive squamous cell carcinomas were distinguished by the depth of penetration into the dermis. Fully invasive squamous cell carcinomas were tumors that invaded the panniculus carnosus. Papillomas were considered benign while microinvasive and fully invasive squamous cell carcinomas were considered malignant. Average malignant tumor percentages were calculated using the total number of graded tumors per treatment group.

### 2.3. Immunohistochemistry

*p53*. Skin sections were examined for epidermal p53 via immunohistochemistry as previously described [10, 11].

*Mutant p53*. After rehydration, slides were incubated in 3% H_2_O_2_ in water for 10 minutes at room temperature to block endogenous peroxidase activity. Slides were subjected to antigen retrieval in a microwave, after which they were blocked with avidin D and biotin (Vector Laboratories), each for 15 minutes, 1× Casein for 30 minutes, and incubated with primary mutant p53 antibody (Novus Biologicals) at a 1 : 3000 dilution in 1× Casein at 4°C overnight. Slides were then incubated with biotinylated F (ab)′ (Accurate Chem) at a 1 : 250 dilution in 1× Casein, followed by ABC Elite. Slides were incubated in DAB solution (Vector Laboratories) for 10 minutes at RT. Positively stained area in the epidermis was examined at 10x and 20x magnification using ImageJ software (NIH).

### 2.4. Statistical Analysis

The results presented in this paper were part of two separate experiments, starting approximately 1 year apart, each involving four treatment groups and a single control group. Considering the results for the 25-week UVB exposure model only, Dunnett's adjustment [12, 13] for multiplicity was used for comparing the primary outcome of tumor burden at 24 weeks between the groups in order to control the probability of a type I error at 5%. The number of control mice was inflated compared to the treatment groups to increase the power of the comparison [12]. Residual plots verified the model assumptions of normality and homoscedasticity and a logarithmic transformation was utilized if necessary. Continuous outcome data were analyzed using an ANOVA approach with linear contrasts for testing the comparisons of interest. For count data, the Poisson regression was used. All analyses were conducted in SAS version 9.2 (SAS Institute, Cary, NC). *P*  values ≤ 0.05 were considered statistically significant. No adjustments were made for multiple comparisons between the studies. The major limitations of comparisons between the two studies are that mice were not randomized to the two exposure protocols and the studies were run at different times. Thus, the exposure effect is aliased with any batch effect of the mice selected for the particular study. However, as Skh-1 mice were used for both studies, we believe any batch effect to be minimal.

## 3. Results

### 3.1. Effects of UVB Exposure Regime Length on Tumorigenesis, Malignancy, and p53 Status in Male and Female Mice

**Figure 1:**
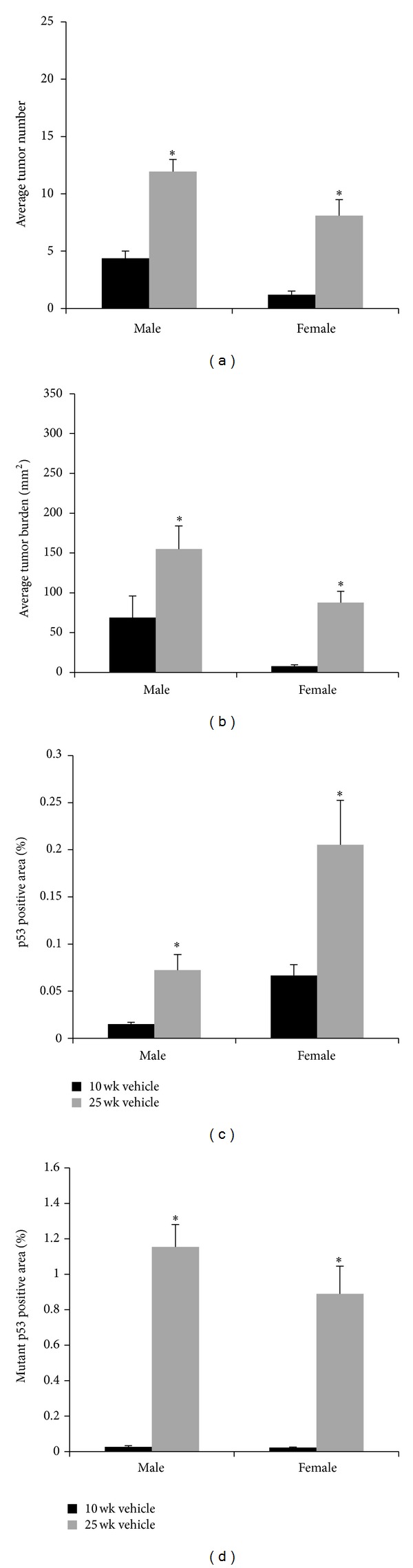
*Effect of length of UVB treatments on tumor number, tumor burden, and p53 status in male and female mice. *(a) Vehicle-treated male and female mice developed more tumors in the 25-week exposure model (*P < 0.0001) compared to the 10-week model. (b) Male (*P = 0.0006) and female (*P < 0.0001) vehicle-treated mice in the 25-week exposure model exhibited increased tumor burden compared to the 10-week model. (c) Male (*P = 0.0002) and female (*P = 0.011) mice treated with vehicle in the 25-week model exhibited elevated epidermal p53 staining compared to the 10-week exposure model. (d) Male and female mice treated with vehicle in the 25-week exposure model exhibited elevated epidermal mutant p53 staining (*P < 0.0001) compared to the 10-week UVB model. Error bars = mean+/−SEM.

To examine the effects of increased UVB protocol length on tumor development, we exposed Skh-1 hairless mice to 2240 J/m^2^ UVB three times weekly for 10 (10-week UVB exposure model) or 25 (25-week UVB exposure model) weeks to model chronic sun exposure. Nonirradiated mice did not develop tumors with any topical treatments. Male mice treated with vehicle in the 25-week UVB exposure model developed a 2.7-fold increase in tumor number (*P* < 0.0001, Figure 1(a)) and a 4.2-fold higher tumor burden (*P* = 0.0006, Figure 1(b)) compared to vehicle-treated mice in the 10-week UVB exposure model. Female mice in the 25-week model treated with vehicle displayed a 6.75-fold increase in tumor number (*P* < 0.0001, Figure 1(a)) and a 15.3-fold increase in tumor burden (*P* < 0.0001, Figure 1(b)) compared to vehicle-treated mice in the 10-week UVB exposure model. 

Tumors were isolated from mice at the end of 25 weeks from both the 10-week and 25-week UVB exposure models, formalin fixed, paraffin embedded, H&E stained, and scored by a board-certified veterinary pathologist (DFK). Male and female mice treated topically with vehicle in the 25-week model developed more malignant tumors per mouse compared to vehicle-treated mice in the 10-week model, with males developing 1.9-fold more malignant tumors and females developing 3.05-fold more tumors (19.3% versus 37.7%, *P* = 0.0467 and 10.3% versus 31.7%, *P* = 0.0319, resp., Table 1). 

As a measure of overall DNA damage, tumor-free, dorsal skin sections were examined for epidermal p53-positive cells via immunohistochemical analysis. Male mice in the 25-week UVB exposure model treated with vehicle exhibited a 3.74-fold increase in p53-positive compared to vehicle-treated mice in the 10-week UVB exposure model (*P* = 0.0002, Figure 1(c)), while females in the 25-week UVB exposure model exhibited a 2.52-fold increase compared to female mice in the 10-week UVB exposure model (*P* = 0.011, Figure 1(c)). 

To further investigate DNA damage, tumor-free, dorsal skin sections were examined for epidermal mutant p53-positive cells via immunohistochemistry. Both male and female mice in the 25-week UVB exposure model treated with vehicle displayed 45.25- and 33.94-fold increased levels of mutant p53-positive cells, respectively, compared to mice in the 10-week UVB exposure model (*P* < 0.0001, Figure 1(d)).

### 3.2. Efficacy of Diclofenac on Tumor Number, Burden, and p53 Status in Male and Female Mice

**Figure 2:**
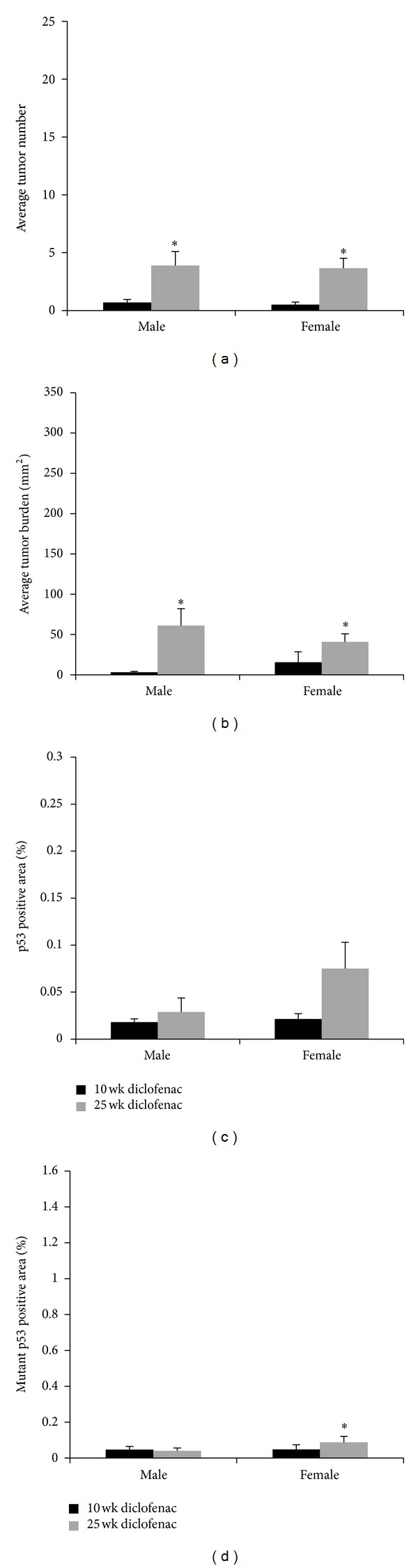
*Efficacy of diclofenac on tumor number, burden, and p53 status in male and female mice. *(a) Male and female diclofenac treated mice developed significantly more tumors in the 25- compared to the 10-week model (*P < 0.0001). (b) Male (*P < 0.0001) and female (*P = 0.0005) diclofenac-treated mice in the 25-week UVB model developed significantly larger tumor burden compared to mice in the 10-week model. (c) Male and female mice treated with diclofenac in the 25-week exposure model did not exhibit significantly altered epidermal p53-positive area compared to the 10-week exposure model. (d) Female mice treated with diclofenac exhibited increased epidermal mutant p53-positive area (*P = 0.0060) in the 25- compared to the 10-week model. Error bars = mean+/−SEM.

To determine the effect of topical application of the anti-inflammatory drug diclofenac on tumor development in both the 10-week and 25-week UVB exposure models, Skh-1 hairless mice were exposed to 2240 J/m^2^ UVB three times weekly for 10 weeks. Mice in the 10-week UVB exposure model were then treated topically with diclofenac for 15 weeks without further UVB exposure whereas mice in the 25-week UVB exposure model continued to be exposed to UVB three times a week and were treated topically with diclofenac immediately after each UVB exposure for the remaining 15 weeks. Compared to male mice in the 10-week UVB exposure model, 25-week male mice displayed a 5.57-fold increase in tumor number (*P* < 0.0001Figure 2(a)) and a 9.52-fold increase in tumor burden (*P* < 0.0001; Figure 2(b)). Female mice in the 25-week UVB exposure model displayed a 7.33-fold increase in tumor number (*P* < 0.0001; Figure 2(a)) and a 7.06-fold increase in tumor burden (*P* = 0.0005; Figure 2(b)). 

Importantly, in the 25-week model, compared to vehicle-treated mice, male mice treated topically with diclofenac developed 77% fewer tumors (*P* < 0.0001, Figures 1(a) and 2(a)) and exhibited an 80% reduction in tumor burden (*P* = 0.0002, Figures 1(b) and 2(b)) while female mice treated topically with diclofenac developed 55% fewer tumors (*P* < 0.0001, Figures 1(a) and 2(a)) and exhibited a 68% reduction in tumor burden (*P* = 0.0186, Figures 1(b) and 2(b)) compared to vehicle-treated mice. These results demonstrate that, even with continued UVB exposure, topical treatment with this anti-inflammatory drug continued to be effective in reducing tumor development. 

However, male mice topically treated with diclofenac in the 25-week exposure model developed 9.79-fold more malignant tumors (5.9% versus 57.9%, *P* = 0.0262) compared to male mice treated with diclofenac in the 10-week model. Female mice treated topically with diclofenac developed 2.43-fold more malignant tumors (25% versus 61.3%) in the 25-week model compared to the 10-week model (Table 1), but due to both the smaller amount of tumors developed and the inherent variable nature of this outbred strain, this difference was not statistically significant (*P* = 0.1565). When compared to vehicle-treated mice, male mice in the 25-week UVB model treated topically with diclofenac exhibited a 53.6% increase in malignancy rate, which, due to the variability observed in this outbred mouse strain, was not statistically significant (*P* = 0.1016). Furthermore, in the 25-week UVB model, female mice treated topically with diclofenac displayed an 89% higher malignancy rate compared to vehicle-treated mice (*P* = 0.0227, Table 1). These data suggest that while topical treatment with the anti-inflammatory compound decreased tumor development, it did not decrease malignancy rates observed with continued UVB exposure.

Male mice treated topically with diclofenac exhibited no significant alterations in epidermal p53-positive area between the two models and although female mice treated topically with diclofenac exhibited a 2-fold increase in p53-positive area, this difference was not statistically significant (*P* = 0.1683, Figure 2(c)). 

Male mice in the 25-week UVB exposure model did not exhibit significantly altered epidermal mutant p53 staining with topical diclofenac treatment compared to male mice in the 10-week exposure model. In contrast, female mice treated with diclofenac in the 25-week exposure model exhibited a 4.58-fold increase in mutant p53-positive cells compared to female mice in the 10-week UVB exposure model (*P* = 0.0060, Figure 2(d)). 

### 3.3. Efficacy of C E Ferulic on Tumor Number, Burden, and p53 Status in Male and Female Mice

**Figure 3:**
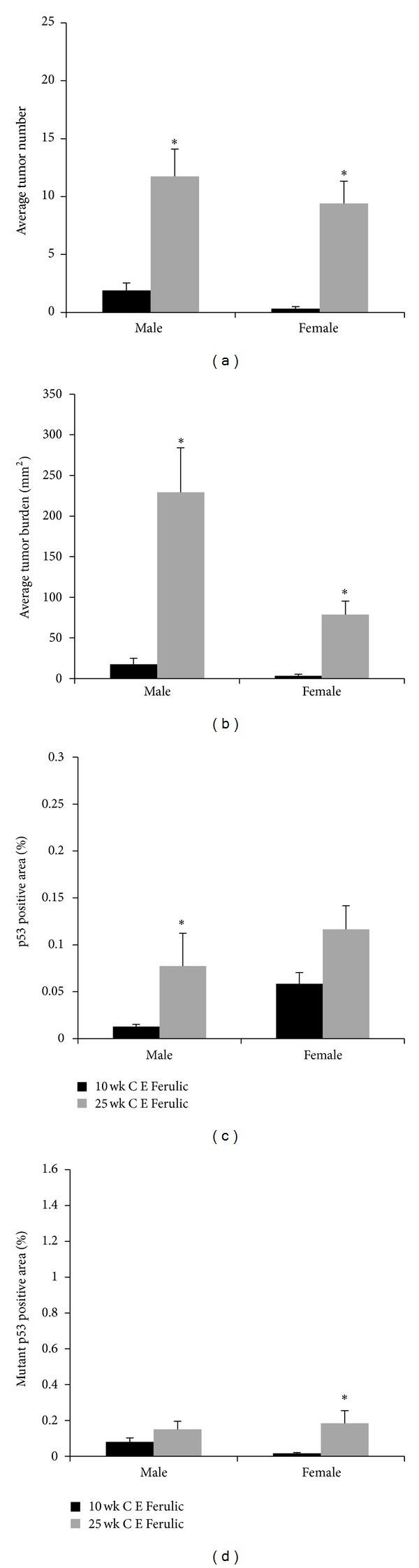
*Efficacy of C E Ferulic on tumor number, burden, and p53 status in male and female mice.* (a) Male and female mice topically treated with *C E Ferulic* developed more tumors in the 25- compared to the 10-week model (*P < 0.0001). (b) Male and female mice topically treated with *C E Ferulic* developed increased tumor burden in the 25- compared to the 10-week model (*P < 0.0001). (c) Male mice treated topically with *C E Ferulic* exhibited increased levels of epidermal p53-positive area in the 25- compared to the 10-week model (*P = 0.0062). (d) Female mice treated topically with *C E Ferulic *exhibited increased levels of epidermal mutant p53-positive area in the 25- compared to the 10-week model (*P = 0.0001). Error bars = mean+/−SEM.

The effect of topical treatment with the stable antioxidant compound C E Ferulic on tumor development was determined in both the 10- and 25-week UVB exposure models by exposing Skh-1 hairless mice to 2240 J/m^2^ UVB three times weekly for 10 weeks. Mice in the 10-week UVB exposure model were then treated topically with C E Ferulic for 15 weeks without further UVB exposure whereas mice in the 25-week UVB exposure model were treated immediately after each UVB exposure for 15 weeks. Male mice in the 25-week UVB exposure model treated topically with C E Ferulic displayed a 5.68-fold increase in tumor number (*P* < 0.0001, Figure 3(a)) and a 30.05-fold increase in average tumor burden (*P* < 0.0001, Figure 3(b)) compared to mice in the 10-week model. Female mice in the 25-week model treated topically with C E Ferulic displayed a 28.2-fold increase in average tumor number (*P* < 0.0001, Figure 3(a)) as well as a 30.69-fold increase in average tumor burden (*P* < 0.0001, Figure 3(b)) compared to mice in the 10-week model. 

Compared to vehicle-treated mice, male mice in the 25-week UVB exposure model treated topically with C E Ferulic did not exhibit a significant alteration in tumor number (Figures 1(a) and 3(a)) but displayed a 60% increase in tumor burden compared to vehicle-treated mice, which possibly due to the variability in this outbred mouse strain did not reach statistical significance (*P* = 0.5590, Figures 1(b) and 3(b)). Female mice in the 25-week UVB model treated topically with C E Ferulic did not exhibit a significant alteration in tumor number (Figures 1(a) and 3(a)) or average tumor burden (Figures 1(b) and 3(b)) compared to vehicle-treated mice.

Isolated tumors were analyzed as described above. Male mice in the 25-week UVB model treated with C E Ferulic developed 3.46-fold more malignant tumors (31.2% versus 45.6%, *P* = 0.0087, Table 1) while female mice treated developed 10.71-fold more malignant tumors (0 versus 31.4%, *P* = 0.0196, Table 1) compared to mice treated with C E Ferulic in the 10-week model. Compared to vehicle-treated mice, neither male nor female mice in the 25-week model treated topically with C E Ferulic exhibited significant alterations in tumor malignancy (*P* = 0.3391 and *P* = 0.7544, respectively, Table 1). These data suggest that topical C E Ferulic treatment is not effective with continued UVB exposure.

Interestingly, male mice treated with topical C E Ferulic exhibited a 3.71-fold increase in epidermal p53-positive cells (*P* = 0.0062, Figure 3(c)) while female mice exhibited no statistically significant alterations in p53-positive area in the 25-week UVB exposure model compared to the mice in the 10-week model (Figure 3(c)). 

Male mice did not exhibit significantly altered epidermal mutant p53 staining with topical C E Ferulic treatment. In contrast, female mice treated with C E Ferulic in the 25-week exposure model exhibited a 8.07-fold increase in mutant p53-positive cells (*P* = 0.0001, Figure 3(d)) compared to female mice treated with C E Ferulic in the 10-week model.

### 3.4. Efficacy of Vitamin E on Tumor Number, Burden, and p53 Status in Male and Female Mice

**Figure 4:**
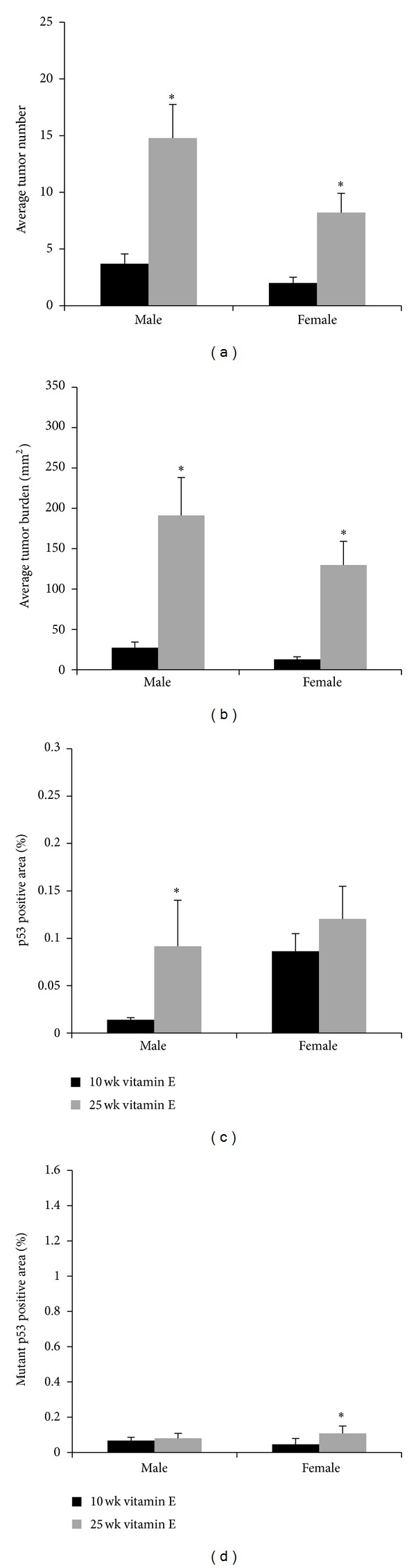
*Efficacy of vitamin E on tumor number, burden, and p53 status in male and female mice.* (a) Male and female mice treated with topical vitamin E developed more tumors in the 25-week compared to the 10-week model (*P < 0.0001). (b) Male and female mice treated with topical vitamin E exhibited increased tumor burden in the 25- compared to the 10-week model (*P < 0.0001). (c) Male mice treated with vitamin E exhibited increased epidermal p53-positive area in the 25- compared to the 10-week model (*P = 0.0023). (d) Female mice treated with vitamin E exhibited significantly increased epidermal mutant p53-positive area in the 25- compared to the 10-week model (*P = 0.0134). Error bars = mean+/−SEM.

The effect of topical treatment with the classical antioxidant vitamin E on tumor development was determined in both the 10- and 25-week UVB exposure models by exposing Skh-1 hairless mice to UVB as described above. Mice in the 10-week UVB exposure model were then treated topically with vitamin E for 15 weeks without further UVB exposure whereas mice in the 25-week UVB exposure model were treated immediately after each UVB exposure for 15 weeks. Male mice in the 25-week model treated topically with vitamin E demonstrated a 3.84 fold increase in tumor number (*P* < 0.0001, Figure 4(a)) and a 9.44-fold increased tumor burden (*P* < 0.0001, Figure 4(b)) compared to mice in the 10-week model. Female mice in the 25-week model treated topically with vitamin E had a similar 4.11-fold increase in tumor number (*P* < 0.0001, Figure 4(a)), and demonstrated a 11.4-fold increased tumor burden (*P* < 0.0001, Figure 4(b)) compared to mice in the 10-week model. 

Compared to vehicle-treated mice, there were no statistically significant differences in tumor number (*P* = 0.0813; Figures 1(a) and 4(a)) or burden (*P* = 0.9100; Figures 1(b) and 4(b)) in male mice treated with vitamin E in the 25-week UVB model. Likewise, compared to vehicle treated mice, topical vitamin E treatment of female mice in the 25-week UVB model did not statistically significantly alter tumor number (*P* = 0.9150; Figures 1(a) and 4(a)) or burden (*P* = 0.7961; Figures 1(b) and 4(b)).

 Isolated tumors were analyzed as described above. Male mice in the 25-week UVB model treated topically with vitamin E developed 4.08-fold more malignant tumors (8.2% versus 34.1%, *P* = 0.0028, Table 1) compared to male mice treated with vitamin E in the 10-week model. Female mice in 25-week UVB model treated topically with vitamin E developed 14.38-fold more malignant tumors (2.9% versus 40.5%, *P* = 0.0087, Table 1) compared to female mice treated topically with vitamin E in the 10-week model. Neither male nor female mice in the 25-week model treated topically with vitamin E exhibited significant alterations in tumor malignancy compared to vehicle-treated mice (*P* = 0.6251 and *P* = 0.2816, resp.). These data demonstrate that with continued UVB exposure, compared to vehicle-treated mice of either sex, topical treatment with vitamin E was not effective in modulating either tumor development or progression.

Interestingly, male mice in the 25-week UVB exposure model treated with topical vitamin E exhibited a 3.92-fold increase in epidermal p53-positive cells compared to the 10-week model (*P* = 0.0023, Figure 4(c)). However, female mice in the 25-week UVB model exhibited no significant alterations in p53-positive area compared to the 10-week model. 

Male mice in the 25-week UVB exposure model treated topically with vitamin E did not exhibit significantly altered epidermal mutant p53 staining compared to those in the 10-week model. In contrast, female mice treated topically with vitamin E in the 25-week UVB model exhibited a 3.78-fold increase in mutant p53-positive cells (*P* = 0.0134, Figure 4(d)) compared to female mice in the 10-week exposure model.

## 4. Discussion

 Epidemiological studies have suggested a correlation between UV exposure and skin cancer development due to a higher frequency of disease among patients with a self-reported history of significant sun exposure. We were interested in determining the extent to which being compliant versus noncompliant with doctors' orders to stay out of the sun could affect cutaneous tumor development in subjects who have accumulated a significant amount of UVB-mediated skin damage over their lifetime. To that end, male and female Skh-1 mice were used to model compliant versus noncompliant patients, that is, to evaluate the effects of 10 weeks versus 25 weeks of UVB exposure on cutaneous tumor number, burden, grade and p53 status. We were also interested in the effect that continued 25-week UVB exposure had on the efficacy of potential topical therapeutic strategies compared to the effect on skin that was chronically UVB damaged but no longer being actively exposed to UVB. In this study where the extent of UVB exposure was directly monitored and recorded, we demonstrated an increased tumor number, burden, and grade at 25 weeks in in both male and female Skh-1 mice exposed to 2240 J/m^2^ UVB three times weekly for 25 weeks compared to 10 weeks. While previous reports have demonstrated that increasing UV doses resulted in a shorter latency period, increased DNA damage and p53 mutations, and increased tumor number, the current study demonstrates that the length of repetitive nonburning amounts of UVB exposure likewise enhances the carcinogenesis process [14, 15]. The increases in tumor burden, tumor grade, and malignancy rate in both male and female mice correlated with increased epidermal p53 and mutant p53 protein in mice exposed to 25 compared to 10 weeks of UVB exposure.

Our previous study demonstrated significantly decreased tumor number and burden in diclofenac-treated mice of both sexes, compared to vehicle-treated 10-week UVB-exposed mice [10]. The current study found that despite the overall increases in tumor number and burden observed in the 25-week model, topical diclofenac treatment continued to effectively decrease tumor number and burden in both male and female mice compared to mice treated with vehicle. In contrast, male and female mice treated with topical antioxidants did not exhibit any beneficial effects in terms of tumor number or burden and in fact demonstrated increases in both parameters. Surprisingly, our previously reported increased malignancy rate in female mice treated topically with diclofenac [10] was exacerbated in the current study, where female mice exposed to UVB for 25 weeks and treated topically with diclofenac exhibited a 90% increase in malignancy rate compared to vehicle-treated mice despite having fewer tumors. Previously we had shown that male mice exposed to UVB and treated with topical diclofenac displayed a decreased malignancy rate compared to vehicle-treated mice [10]. However, with 25 weeks of UVB exposure, male mice treated with diclofenac actually exhibited a 50% increase in malignancy rate compared to vehicle-treated mice. The unexpected observed increases in malignancy rates for both sexes in the 25-week UVB exposure model suggest that the more benign tumors may be more affected by the topical treatment while the malignant tumors are escaping the chemopreventive treatment by an as of yet unexplained mechanism. Many previous studies that examine possible therapeutics or preventative treatments for cancers examine the number and size of tumors that appear but do not report the histologic grade of tumors that did develop, especially if they are small. Our current studies highlight the importance of histologically examining all tumors, including those that develop in groups where treatment may have been effective in decreasing tumor number or burden but may be not effective against eradicating/preventing the more malignant tumors.

 We previously demonstrated that the combination of the antioxidants vitamin E, vitamin C, and ferulic acid found in C E Ferulic exerted potential benefits in terms of decreased tumor number and burden in both male and female mice. In contrast, while moderate benefits were observed with vitamin E topical treatment alone in male mice, female mice treated topically with vitamin E exhibited significantly increased overall DNA damage, cutaneous proliferation, and angiogenesis, as well as increased tumor growth rate, number, and burden [11]. The current study demonstrated that, with continued, prolonged chronic UVB exposure, any observed benefits of topical antioxidant treatment were lost in both sexes. These data highlight not only the detrimental effects of continued UVB exposure on ultimate tumor development and progression but also the importance of limiting UVB exposure to obtain the optimal efficacy of therapeutic interventions. 

## 5. Conclusions

 These studies illustrate that, in terms of tumor number and burden, both males and females benefit from topical diclofenac treatment, regardless of overall length of UVB exposure regime. However, especially in females, the elevated malignancy rate of mice treated topically with diclofenac may overshadow the benefits. Further, while we previously demonstrated potential benefits of combination antioxidant topical treatment for decreasing tumor burden and tumor malignancy rates [11], these effects were lost in the 25-week UVB exposure model, indicating that sun exposure must be limited in both males and females in order to benefit from antioxidant treatments. Overall, these data support the commonly assumed, but not demonstrated, fact that cumulative length of UVB exposure is a risk factor for UVB-induced SCC and highlight the fact that changing sun worshiping habits, even after early chronic sun exposure and skin damage, may ultimately decrease tumor development in patients.

## Figures and Tables

**Table 1:** Average percentage of malignant tumors per treatment group after 10 and 25 weeks of UVB exposure in male and female mice.

		10-week UVB	25-week UVB	*P* value (25 weeks Tx versus Veh)	*P* value (10 weeks versus 25 weeks)
Male	UVB/vehicle	19.3	37.7	—	0.0467*
	UVB/diclofenac	5.9	57.9	0.1016	0.0262*
	UVB/CE	31.2	45.6	0.3391	0.0087*
	UVB/vitamin E	8.2	34.1	0.6251	0.0028*

Female	UVB/vehicle	10.3	31.7	—	0.0319*
	UVB/diclofenac	25.0	61.3	0.0227*	0.1565
	UVB/CE	0.0	31.4	0.7544	0.0196*
	UVB/vitamin E	2.9	40.5	0.2816	0.0087*

*: The comparison of the percentage of malignant tumors in male mice treated with vehicle in the 25 weeks compared to the 10 weeks of UVB exposure model. For clarity, all of the *P* values in the final column of Table 1 refer to 25 versus 10 weeks of tumor malignancy within a treatment group and gender.
